# Mediators linking insecure attachment to eating symptoms: A systematic review and meta-analysis

**DOI:** 10.1371/journal.pone.0213099

**Published:** 2019-03-07

**Authors:** Laura Cortés-García, Bahi Takkouche, Gloria Seoane, Carmen Senra

**Affiliations:** 1 Department of Clinical Psychology and Psychobiology, University of Santiago de Compostela, Santiago de Compostela, Galicia, Spain; 2 Department of Preventive Medicine, University of Santiago de Compostela, Santiago de Compostela, Galicia, Spain; 3 Centro de Investigación Biomédica en Red de Epidemiología y Salud Pública (CIBER-ESP), Madrid, Spain; 4 Department of Social Psychology, Basic and Methodology, University of Santiago de Compostela, Santiago de Compostela, Galicia, Spain; Chiba Daigaku, JAPAN

## Abstract

In the last two decades, the number of studies focused on the mediators connecting insecure attachment with Eating Disorders (EDs), at both clinical and sub-clinical level, has considerably increased. However, there has not been a systematic synthesis of this literature to date. To fill this gap, the current meta-analytic review aimed at identifying and quantifying the extent to which mediators contribute to the explanation of this relationship. The present study was registered with PROSPERO (CRD42017076807). A comprehensive search process in seven different electronic databases retrieved 24 studies that examined how insecure attachment leads to ED symptoms through mediation analysis. Standardized regression coefficients of the indirect and total paths of 21 mediation models were pooled. Studies were coded and ranked for quality. We found evidence to show that maladaptive emotion regulation and depressive symptoms had the highest effect size for mediation (mediation ratio [*P*_*M*_] = 0.71). Further, body dissatisfaction, neuroticism, perfectionism, mindfulness and social comparison had significant, but moderate to low mediating effects (*P*_*M*_ = 0.21–0.58). The methodological quality of these studies was mostly low to moderate and potential areas for development were highlighted. Our findings support the direct targeting of these psychological constructs in prevention programs and treatment of EDs. Future investigations addressing the time sequence between the variables will provide valuable clues to untangle the prospective contribution of each variable on the development and maintenance of eating pathology.

## Introduction

Broad research has found evidence of the relationship between insecure attachment style and mental disorders, including Eating Disorders (EDs) [1–3]. It is accepted that individuals with EDs have a higher prevalence of insecure attachment than healthy controls as it has been unanimously established in previous reviews [3–6]. In addition, insecure attachment seems to be a risk factor for the subsequent emergence of disordered eating in non-clinical populations [7,8].

In the last two decades several researchers have explored the connection between insecure attachment and eating pathology at both clinical and sub-clinical level, in cross-sectional [9,10] and longitudinal studies [11,12]. Identifying the mechanisms by which insecure attachment may increase the vulnerability to EDs across the life span may offer novel perspectives for clinical assessment, prevention and treatment [7,13]. Nevertheless, despite the great interest and growing number of studies on this topic, to date there has not been any systematic synthesis of this literature.

### Insecure attachment and vulnerability to eating pathology

Attachment is an emotional long-lasting bond that infants develop with the main caregivers over the first years of life [14]. Based on this early caregiving environment, children develop representations about themselves and about the social world, known as *Internal Working Models*, which will guide their psychosocial functioning across life span [15,16]. When primary attachment figures are emotionally available, consistent and responsive to the child’s needs, they provide a solid base for exploring the world, where the *self* is viewed as valued and loved and, represent a *safe haven* where the child can reach comfort and protection in times of distress ([17–19]. Repetitive attachment-related experiences with a sensitive caregiver also contribute to the development of adaptive emotion regulation skills [20]. In contrast, insecure attachment relationships do not provide stable assistance and support to facing threating situations [21]. As a result, insecurely attached children develop dysfunctional cognitive patterns alongside with inconsistent emotion regulation abilities that lead them to interact either restricting (avoidant attachment) or exaggerating (anxious attachment) their need for comfort, security and proximity to their attachment figures [14,20]. In this respect, anxiety and avoidance can be conceptualized as two dimensions aligned as opposite ends of a continuum in which different styles of insecure attachment can be located [22,23].

Broad research guided by the theory of attachment has established clear associations between insecure attachment style and the expression and maintenance of unhealthy eating attitudes and behaviors, at both clinical and sub-clinical level [5,7,8]. Nevertheless, the literature inquiring into the association between different attachment styles and specific ED subtypes remains inconclusive [3,7]. Consequently, current research tends to examine the link between insecure attachment (regardless of a specific style) and the severity of eating symptomatology across diagnosis [2,13]. Similarly, in non-clinical samples there is evidence for the association between insecure attachment and premorbid symptoms such as higher weight concerns [24], body dissatisfaction [25,26], restrictive eating [27] and binge eating [28] supporting therefore the hypothesis that eating disorder symptoms can be the manifestations of the psychological and emotional processes developed from insecure attachment relationships.

However, an insecure attachment is not a sufficient cause for the development of psychopathology [19], but in most cases this influence may be indirectly conditioned by the concurrence of different variables that can modify the early attachment experiences [29,30]. In this regard, research suggests that the relationship between insecure attachment and eating disorder symptoms may be explained by multiple mediating mechanisms [3,19]. In particular, studies hint towards the potential role of problematic emotional regulation, negative affect, maladaptive perfectionism and body dissatisfaction explaining how attachment insecurity might put someone at risk for ED at young age [8,31] or might maintain ED symptoms [5,13]. The understanding of these intermediate variables driving from early insecure experiences to the symptomatic expression of eating pathology across time provides new insight into this topic.

As noted previously, insecurely attached people are more likely to report negative feelings about the self, experience interpersonal difficulties and use maladaptive coping strategies, such as suppress/avoid (i.e., deny stress or divert attention, consistent with avoidant style) or accentuate emotional experiences (i.e., rumination or self-blame, consistent with anxious style), to deal with distress [21,32]. Collectively, it can be understood that insecurely attached people in their hopes to reduce negative feelings about themselves turn to eating symptoms such as dieting (to reach perfect body image), binge eating (to increase mood and get distracted) and purging (to compensate the negative feelings of breaking the diet and avoid gaining weight) [21,33,34]. Due to the lack of functionality of these strategies, negative affect remains and keeps the cycle activated, triggering the symptomatology every time the person has to face distress [7].

### Previous reviews and aims for the current study

Since the publication of the review by Ward et al. [6], the number of studies focused on the mechanisms whereby the insecure attachment might trigger the development of eating symptomatology has considerably increased. In fact, some recent research pointed out to specific mechanisms linking insecure attachment and the expression of disordered eating that shed light into the processes involved in the development of the symptoms [8,13].

On the other hand, statistical analyses used to determine how or why an independent variable transmits its effect on a dependent variable, namely the hypothesis of mediation, have experienced an important development in the last 20 years [35–37].

In spite of the advances and increased interest about the mediating mechanisms through which insecure attachment confers vulnerability to the development of ED symptoms, no study has systematically reviewed or measured the impact of these mediators to date [3,7]. In order to overcome these gaps, we aim at identifying the pathways through which insecure attachment may lead to eating psychopathology and at quantifying the size effect of the mediators through a meta-analysis. Taking into account the prior literature, we expect that emotional dysregulation and depressive symptoms along with other psychological variables, such as body dissatisfaction and perfectionism, will mediate the relation between attachment and ED psychopathology. This knowledge not only will contribute to a better understanding about specific factors that could play a role in the maintenance of EDs, but will also provide empirical evidence for existing theories and for formulation of new hypotheses for treatment targets [7,38,39].

## Method

### Registration of systematic review and meta-analysis

The protocol for the present study was registered with the PROSPERO international prospective register of systematic reviews (CRD42017076807) and can be accessed at http://www.crd.york.ac.uk/PROSPERO/display_record.php?ID=CRD42017076807. Our review is reported in accordance with the PRISMA [40] (S1 Table).

### Search strategy

A systematic literature search was performed on the following databases: Medline, Pubmed, WOS (Web of Science), Scopus, PsycINFO, EMBASE and Conference Proceedings Citation Index-Science (CPCI-S) and Social Science & Humanities (CPCI-SSH) up to January 2019. The initial search terms used as keywords were: [(attachment style OR attach*) AND (eating disorder OR eating symptom*)]. Besides, to focus the search identifying studies reporting on mediational mechanisms, a tailored search strategy was conducted: [(attachment style OR attach* OR attachment) AND (eating disorder* OR eating symptom* OR eating psycho* OR "disordered eating") AND (mediat* OR indirect OR "structural equation modeling" OR "structural equation modelling" OR "SEM" OR (Baron AND Kenny) OR Mackinnon OR "product of coefficient" OR "difference in coefficient" OR sobel OR "causal pathway" OR intermediate OR "indirect effect" OR mechanism)]. Reference lists of included studies, as well as recent reviews in the field, were scanned for any additional relevant studies.

### Inclusion/exclusion criteria

For inclusion in this study, the studies had to meet the following criteria: (1) published in a peer-reviewed journal prior to January 2019; (2) reported on mediators between attachment style (towards physical person) and eating disorder symptoms; (3) used standardized measures (either through self-report, administered questionnaires or structured interviews) of attachment style, eating pathology and mediating variables such as: ECR (Experiences in Close Relationships), EAT (Eating Attitudes Test), EDI (Eating Disorder Inventory) or EDEQ (Eating Disorder Examination Questionnaire); (4) used a formal mediation analysis (e.g. Baron and Kenny’s causal steps of mediation, structural equation modelling) or significance tests of mediation (e.g. Sobel test, bootstrapping); (5) carried out with participants at any age from clinical (by criteria DSM-IV, DSM-IV-TR, or DSM-5) or non-clinical samples (individuals reporting symptoms of EDs but who endorse subthreshold levels of one or more symptoms [41]) and (6) written in English, Spanish, German or French. In order to perform the meta-analysis, additionally to these inclusion criteria, we required at least two studies per mediator.

Patients with obesity and comorbid ED were excluded. We did not consider papers exploring perceived quality of relationships or attachment as a mediator since the focus of our study is to understand attachment as an independent variable.

### Study selection

**Fig 1:**
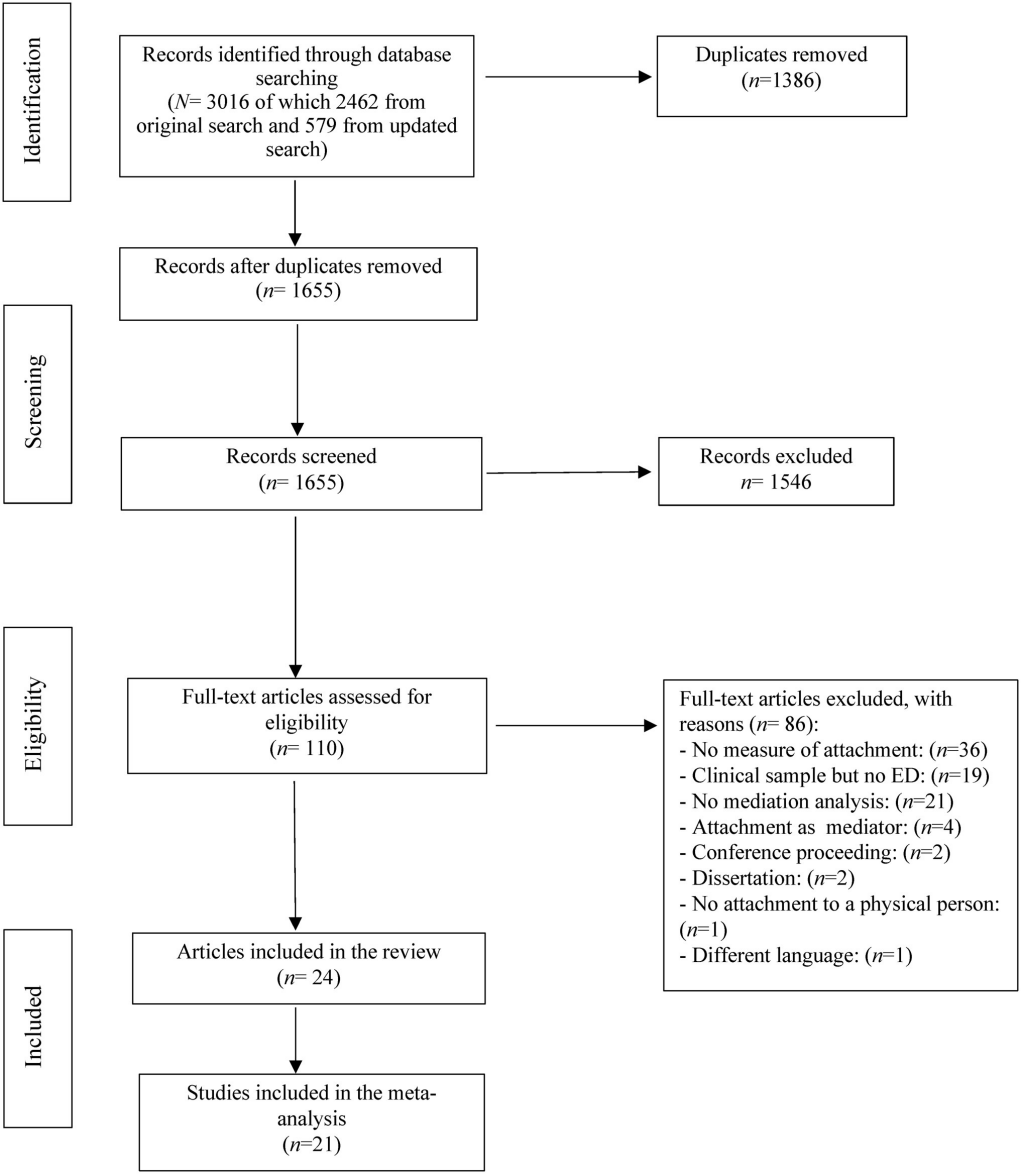
Flowchart for search strategy. Fig 1 represents the flow information through the different four phases of the systematic review. Following PRISMA guidelines, a total of 24 studies were eligible to be included in the systematic review and 21 model were included to be meta-analyzed.

Preliminary screening of the studies obtained by the systematic search was performed by the main author (LCG). Two co-authors (LCG and CS) reviewed all titles and abstracts, excluded studies that did not address mediational analysis, and independently examined each full article to determine final inclusion or exclusion. Reasons for exclusion of full texts were recorded and documented in a PRISMA flow diagram (Fig 1). Discordances on inclusion or exclusion of articles were analyzed, and disagreements were resolved via discussion.

### Data extraction

Two data extraction templates were specifically developed for the current review. First, basic descriptive information about the study was collated (Tables 1 and 2). A second template was used to extract the required data for the meta-analysis. The data extracted and coded are displayed in S2 Table. Up to 3 e-mail requests were also sent to the corresponding author to obtain any unpublished data necessary to perform the meta-analysis [11,42–45]. In case the corresponding author did not respond, a subsequent email was sent and copied to a second author. Where this failed to generate a response, attempts to obtain the data were terminated.

**Table 1:** Summary of reviewed studies with non-clinical samples.

Author/s(year) Country	Sample characteristics (*n*; *M*_age_, *SD*; % female)	Design and mediation test	Attachment figure	Attachment measures	Mediator measures	Outcome measures	Attachment type	Mediation results	Quality rating
Dakanalis et al. (2016) Italy [11]	2055 College students; *M*_age_ = 18.34, *SD* = 0.28; 52,2%	LO; SEM Bootstrapping	Close relationships	ASQ	16-item Hypersensitive Narcissism Scale of NPI-40 (Italian version)	Dieting and Bulimia subscales of EAT-26	Anxious, Avoidant	Vulnerable narcissism fully mediated the effect of attachment anxiety on future bulimic behaviors among women and men. Grandiose narcissism fully mediated the association between attachment avoidance and future dieting behaviors in women and in men.	Moderate
Boone (2013) Belgium [28]	328 Adolescents; *M*_age_ = 17.1, *SD* = 1.13; 57%	CS; Baron & Kenny steps, Sobel test	Mother	ECR-R	PSP subscale of PSPS; SPP subscale of MPS-H&F	Bulimia subscale of EDI-II	Avoidant	Perfectionism Self-Promotion fully mediated the relationship between avoidant attachment towards father and binge eating.	Moderate
Bäck (2011) Sweden [42]	80 High school students; *M*_age_ = 18, *SD* = 0.62; 43,75%	CS; Hierarchical regression analysis, Sobel Test	Mother	AAP	Body and weight dissatisfaction through 2 items	ChEAT	Fearful	Body and weight dissatisfaction fully mediated the relationship between secure mother attachment and eating problems. Body and weight dissatisfaction partially mediated the relationship between fearful mother attachment and eating problems.	Weak
Bamford & Halliwell (2009) UK [43]	213 Undergraduate students; *M*_age_ = 21, *SD* = 4.1; 100%	CS; SEM	Close relationships	ECRQ-R	SCMPS	EDI	Anxious	Social comparison mediated the relationship between insecure attachment and ED.	Weak
Eggert, Levendosky & Klump (2007) US [46]	85 Twins and triplets’ community/university; *M*_age_ = 20.6, *SD* = 2.7; 100%	CS; Hierarchical linear models regressions	Romantic partner	AAS	NEO-PI-R	MEBS	Anxious	Neuroticism fully mediated the relationship between insecure attachment and eating symptoms.	Weak
Han & Pistole (2014) US [47]	381 University students; *M*_age_ = 25.29, *SD* = 6.0; 58%	CS; SEM	Close relationships	ECR-S	DERS	BES	Insecure	Attachment insecurity and binge eating were associated and mediated by emotion dysregulation.	Moderate
Kiang & Harter (2006) US [48]	146 Undergraduate students; *M*_age_ = 19.5; 100%	CS; SEM	Close relationships, Mother	ECR	Ineffectiveness, perfectionism, interpersonal distrust, interoceptive awareness, and maturity subscales from EDI-2	Drive for thinness and bulimia subscales from EDI-2	Anxious, Avoidant	Eating psychological symptoms mediated the relationship between insecure attachment and eating behavioural symptoms.	Weak
Koskina & Giovazolias (2010) Greece [49]	481 Undergraduate students: Male: *M*_age_ = 21.92, *SD* = 3.75; Female: *M*_age_ = 20.75, *SD* = 3.50; 79.2%	CS; Baron & Kenny steps, Sobel test	Romantic partner	ECR-SR	BSQ-34	EAT-26	Anxious	Body dissatisfaction fully mediated the relationship between insecure anxious attachment and eating symptomatology (dietary symptoms and bulimia) among women. Body dissatisfaction mediated the relation between anxious attachment and dietary among men.	Weak
McDermott et al. (2015) US [50]	2644 University students; *M*_age_ = 22.5, *SD* = 5.26; 46%	CS; SEM Bootstrapping	Romantic partner	ECR-S	ATHS	CCAPS-62	Anxious, Avoidant	Hope mediated the associations between adult attachment dimensions (anxiety and avoidance) and eating problems.	Moderate
Pepping et al. (2015) Australia [51]	Study 1: 144 Undergraduate students; *M*_age_ = 20.89, *SD* = 4.80; 100%	CS; Bootstrapping	Close relationships	ECR-R	FFMQ	EDI-3	Anxious, Avoidant	Lower mindfulness mediated the associations of both attachment anxiety and avoidance with increased eating pathology.	Weak
Schembri & Evans (2008) Australia [52]	225 Participants community/university; *M*_age_ = 30.24, *SD* = 10.44; 100%	CS; Hierarchical Multiple Regression, Sobel Test	Romantic partner	Anxiety dimension from ECR	CESD, RSE	BULIT-R	Anxious	General psychopathology (combined scores of depression and self-esteem) partially mediated the relationship between anxious attachment and bulimic symptoms.	Weak
Shanmugan, Jowett & Meyer (2012) UK [53]	411 British athletes; *M*_age_ = 20.95, *SD* = 3.67; 61,3%	CS, Bootstrapping	Mother, Father, Peers, Coach	ECR	FMPS, DAS, RSES, Subscale of depression of SCL-90	EDEQ	Anxious, Avoidant	Self-esteem, depression, and self-critical perfectionism mediated between Insecure attachment styles (anxious and avoidant) and athletes’ eating pathology.	Moderate
Ty & Francis (2013) Australia [54]	247 Participants community; *M*_age_ = 24.51, *SD* = 4.05; 100%	CS, Bootstrapping	Close relationships	ECR-RS	SCMPS, PACS, DERS	EAT-26	Anxious, Avoidant	Social comparison and emotion dysregulation mediated between insecure attachment and disordered eating.	Weak
Van Durme, Braet & Goossens (2015) Belgium [55]	952 Adolescents primary/secondary schools; *M*_age_ = 12.9, *SD* = 1.06; 54,6%	CS; Bootstrapping	Mother	ECR-R-C	FEEL-KJ	ChEDE-Q	Anxious, Avoidant	Maladaptive emotion regulation partially mediated the relationships between both attachment anxiety and attachment avoidance towards mother and restraint and eating pathology concerns.	Moderate

*Note*. CS = Cross-sectional; LO = Longitudinal; SEM = Structural Equation Modelling; AAP = Adult Attachment Protypes; ChEAT = Children’s Eating Attitudes Test; ECRQ-R = Experiences in close relationships questionnaire-Revised; SCMPS = Social Comparisons to models and peers scale; EDI = Eating Disorder Inventory; ECR-R = Experiences in Close Relationships Scale-Revised; PSPS = Perfectionistic Self-presentation Scale; MPS-H&F = Multidimensional Perfectionism by Hewitt and Flett; EDI-II = Eating Disorders Inventory-Two; ASQ = Attachment Style Questionnaire; NPI = Narcissistic Personality Inventory; EAT-26 = Eating Attitude Test-26; NEO-PI-R = NEO Personality Inventory-Revised; MEBS = Minnesota Eating Behavior Survey; DERS = Difficulties in Emotion Regulation Scale; BES = Binge Eating Scale; ECR = Experiences in Close Relationships Scale; EDI-2 = Eating Disorders Inventory-Two; ECR-SR = Experiences in Close Relationships Scale-Revised; BSQ-34 = Body Shape Questionnaire-24; ATHS = Adult Trait Hope Scale; CCAPS-26 = Counseling Center Assessment of Psychological Symptoms-62; FFMQ = The Five Facet Mindfulness Questionnaire; EDI-3 = Eating Disorders Inventory-Three; CESD = Centre for Epidemiological Studies—Depression Scale; RSE = Rosenberg Self-Esteem Scale; BULIT-R = Bulimia Test—Revised; ECR-RS = Relationships Structures; FMPS = Frost Multidimensional Perfectionism Scale; DAS = Dysfunctional Attitude Scale; RSES = Rosenberg’s Self-Esteem Scale; SCL-90-R = Symptom Checklist 90-Revised; PACS = Physical appearance comparison scale; ECR-R-C = The experiences of Close Relationships-Revised-Child Version; FEEL-KJ = The questionnaire to assess children’s and adolescents’ER strategies; ChEDE-Q = The children’s eating disorder examination-questionnaire.

**Table 2:** Summary of reviewed studies with clinical samples.

Author/s(year) Country	Sample characteristics (*n*; *M*_age_, *SD*; % female)	Design and mediation test	Attachment figure	Attachment measures	Mediator measures	Outcome measures	Attachment type	Mediation results	Quality rating
Tasca et al. (2009) Canada [44]	310 Patients with ED (AN = 74, BN = 138, EDNOS = 98); BMI = 21.88 (6.20); *M*_age_ = 26.31, *SD* = 8.76; 100%	CS; SEM, Bootstrapping	Close relationships	ECR	DSI-R, PAI	EDI	Anxious	Emotional reactivity mediated the relationship between attachment anxiety contributed and both depressive symptoms and ED symptoms. Emotional deactivation did not mediate the relationship between avoidance attachment and ED symptoms.	Moderate
Tasca et al. (2006) Canada [45]	268 Patients with ED (ANR = 30 *M*_age_ = 26.4, *SD* = 10.4; ANB = 43, M_*age*_ = 29.8, *SD* = 10.8; BN = 57, *M*_age_ = 29, *SD* = 8.0; BED = 115, *M*_age_ = 42.3, *SD* = 10.8; EDNOS = 23 *M*_age_ = 26.2, *SD* = 8.6); 100%	CS; SEM	Close relationships	ASQ	Body dissatisfaction scale-EDI, Body Esteem-Appearance, Body esteem-Weight scales- BESAA.	Pressure to diet by family of origin, pressure to diet by current relationships, pressure to diet by authority subscales from DSED-R, Drive for thinness scale-EDI, Restraint scale-EDEQ	Anxious, Avoidant	Body dissatisfaction mediated the relationship between attachment insecurity style and restrained eating	Weak
Pepping et al. (2015) Australia [51]	Study 2: 55 Patients with ED (BN = 11, BED = 18, EDNOS = 26); *M*_age_ = 39, *SD* = 12.66; 100%	CS, Bootstrapping	Close relationships	ECR-R	FFMQ	EDI-3	Anxious, Avoidant	Lower mindfulness mediated the associations of both attachment anxiety and avoidance with increased eating pathology	Weak
Dakanalis et al. (2014) Italy [56]	403 Patients with ED (AN = 101, BN = 167, EDNOS = 135); BMI = 17 (0.9); *M*_age_ = 25.33, *SD* = 6.11; 100%	CS; SEM, Bootstrapping	Close relationships	ASQ	MPS	EDI-2	Anxious, Avoidant	Maladaptive perfectionism mediated between both insecure attachment patterns and ED symptoms. It also interacted with insecure attachment to predict higher levels of ED symptoms (moderation).	Moderate
De Paoli et al. (2017b) Australia [57]	744 Participants; *M*_age_ = 22.53, *SD* = 7.60; Clinical group: 122 Patients with ED (AN-R = 56, AN-C = 17, BN = 17, BED = 10, OSFED = 22); BMI = 20.98 (4.59); *M*_age_ = 25.16, *SD* = 7.60; 98% Control group: 662 Participants community/university; *M*_age_ = 22.01, *SD* = 8.63; 79%	CS/Case-control; SEM	Close relationships	ECR-R	RSQ, Appearance RS-Scale, SCRS	EDI-3	Avoidant, Anxious	For the ED group, appearance-based RS and social rank were significant mediators of the relationship between insecure attachment and disordered eating. For the controls, interpersonal RS, appearance-based RS and social rank were mediators of the relationship between insecure attachment and disordered eating.	Moderate
De Paoli et al. (2017a) Australia [58]	616 Participants; *M*_age_ = 22.18, *SD* = 7.77; Clinical group: 108 Patients with ED (AN-R = 50, AN-BP = 15, BN = 17, OSFED = 19); BMI = 21.74 (4.23); *M*_age_ = 25.45, *SD* = 7.65; 100%; Control group: 508 Participants community/university; *M*_age_ = 21.49, *SD* = 7.63; 100%	CS/Case-control; SEM	Close relationships	ECR-R	RSQ, Appearance RS-Scale, SCRS	EDE-Q	Anxious, Avoidant	Emotional deprivation, fear of abandonment, interpersonal RS, and appearance‐based RS mediated between anxious attachment and disordered eating.	Moderate
Jakovina et al. (2018) Croatia [59]	100 Participants; *M*_age_ = 20.40, *SD* = 3.26; 100%; Clinical Group BN = 50; Control group = 50	CS; Multiple regression, Sobel test	Close person	ECR-R	DERS (Difficulties in Emotion Regulation Scale)	EDI-2	Anxiety	Emotion regulation fully mediated between anxious attachment and BN symptoms.	Moderate
Monteleone et al. (2017) Italy [60]	230 Participants; Clinical group:113 Patients with ED (AN = 71; *M*_age_ = 24.7, *SD* = 7.8; BN = 52); BMI = 18.5–25.0; *M*_age_ = 27.8, *SD* = 9.4;100%. Control group: 117 University students; *M*_age_ = 24.7, *SD* = 3.1; 100%	CS/ Case-control; PROCESS, Sobel test, Bootstrapping	Close relationships	ECR-R	IDEA	EDI-II	Avoidant	Embodiment mediated the relationship between avoidant attachment style and ED symptomatology.	Moderate
Monteleone et al. (2018) Italy [61]	123 Participants; Clinical Group = 78, AN R = 38, AN BP = 10; M_age_ = 25.15, *SD* = 9; BN = 30; M_age_ = 27, *SD* = 9.13; Control group = 45, M_age_ = 26.25, *SD* = 1.95; 100%	CS; PROCESS, Bootstrapping	Close person	ASQ	BIS-BAS	EDI-2	Anxiety	Sensitivity to punishment fully mediated the association between anxious attachment style and ED symptoms (drive to thinness and body dissatisfaction).	Moderate
Münch, Hunger & Schweitzer (2016) Germany [62]	253 Participants; *M*_age_ = 25.72, *SD* = 8.73; Clinical group:106 Participants with ED (Self-reported having ED: AN = 45, BN = 29, Other ED = 12; cut-off < 20); *M*_age_ = 24.74, *SD* = 7.71; 100% Control group: 147 Participants; *M*_age_ = 25.72, *SD* = 8.73; 100%	CS/ Case-control; Bootstrapping	Close relationships	AAS	B5T, EXIS	EDE-Q, SEED	Insecure	Personality variables (neuroticism and introversion) and family dysfunction were found to partial mediate the relationship between insecure attachment and eating disorder.	Weak
Redondo & Luyten (2018) Spain [63]	361 Participants; Clinical Group AN = 38; M_age_ = 21.9, *SD* = 5.30; Control group = 327; M_age_ = 19.1, *SD* = 1.94; 100%	CS; SEM	Parents	CaMir	MAAS	EAT-26	Preocupationn, Parental interference, Self-Sufficiency, Childhood trauma	Impairments in cognitive attention to internal mental states mediated the relationship between insecure attachment styles and ED symptoms (Dieting, Bulimia and Food preoccupation, Oral control).	Moderate

*Note*. CS = Cross-sectional; LO = Longitudinal; SEM = Structural Equation Modelling; AN = Anorexia Nervosa; BN = Bulimia Nervosa; BED = Binge Eating Disorder; EDNOS = Eating Disorder Non Otherwise Specified; ASQ = Attachment Style Questionnaire; MPS = Multidimensional Perfectionism Scale; EDI-2 = Eating Disorders Inventory-Two; ECR-R = Experiences in Close Relationships Scale-Revised; RSQ = Rejection Sensitivity Questionnaire; RS = Rejection Sensitivity; SCRS = Social Comparison Rating Scale; EDI-3 = Eating Disorder Inventory-3; EDE-Q = Eating Disorder Examination Questionnaire; DERS = Difficulties in Emotion Regulation Scale; IDEA = Identity and Eating Disorders; BIS-BAS = Behavioral Inhibition System- Behavioral Activation System Scale; B5T = Big-Five Personality Test; EXIS = Experiences in Personal Social Systems Questionnaire; EDEQ = Eating Disorder Examination Questionnaire; SEED = Short evaluation of Eating Disorders; FFMQ = The Five Facet Mindfulness Questionnaire; CaMir = Cartes, Modèles Individuels de Relation; MAAS = Mindfulness Attention and Awareness Scale; BESAA = Body Esteem Scale for Adolescents and Adults; DSED-R = Diagnostic Survey of Eating Disorders-Revised; DSI-R = Differentiation of self-inventory—revised; PAI = Personality assessment inventory; EDI = Eating Disorder Inventory.

### Data synthesis and analysis

Meta-analysis was conducted for a subset of included mediation models using the software Comprehensive Meta-Analysis (CMA). Initial meta-analyses were conducted in order to: 1) calculate the overall effect size of the mediators among all studies, 2) compare if the effect sizes differed according to the attachment type, and 3) investigate if the magnitude of the effect sizes of the mediators differed by gender. Before pooling, we categorized the data by mediators. We used *β* (standardized regression coefficients) or, failing that, *r* (Pearson correlation coefficient) [64], and sample sizes to calculate a pooled effect size for the indirect (a*b) and total effects (path c) [65]. In cases where studies presented their results under the form of unstandardized regression coefficient, we calculated *β* [*β* = B*(*S*_*x*_/*S*_*y*_)] [66]. To provide a summary of each mediation model, we calculated the *mediation ratio* [67] of the pooled indirect effect and the pooled total effect [(*P*_*M*_ = (a*b/c)] [68].

The I^2^ statistic was used to assess the heterogeneity. We present both fixed-effects and random-effects pooled estimates but use the latter when heterogeneity was present.

**Fig 2:**
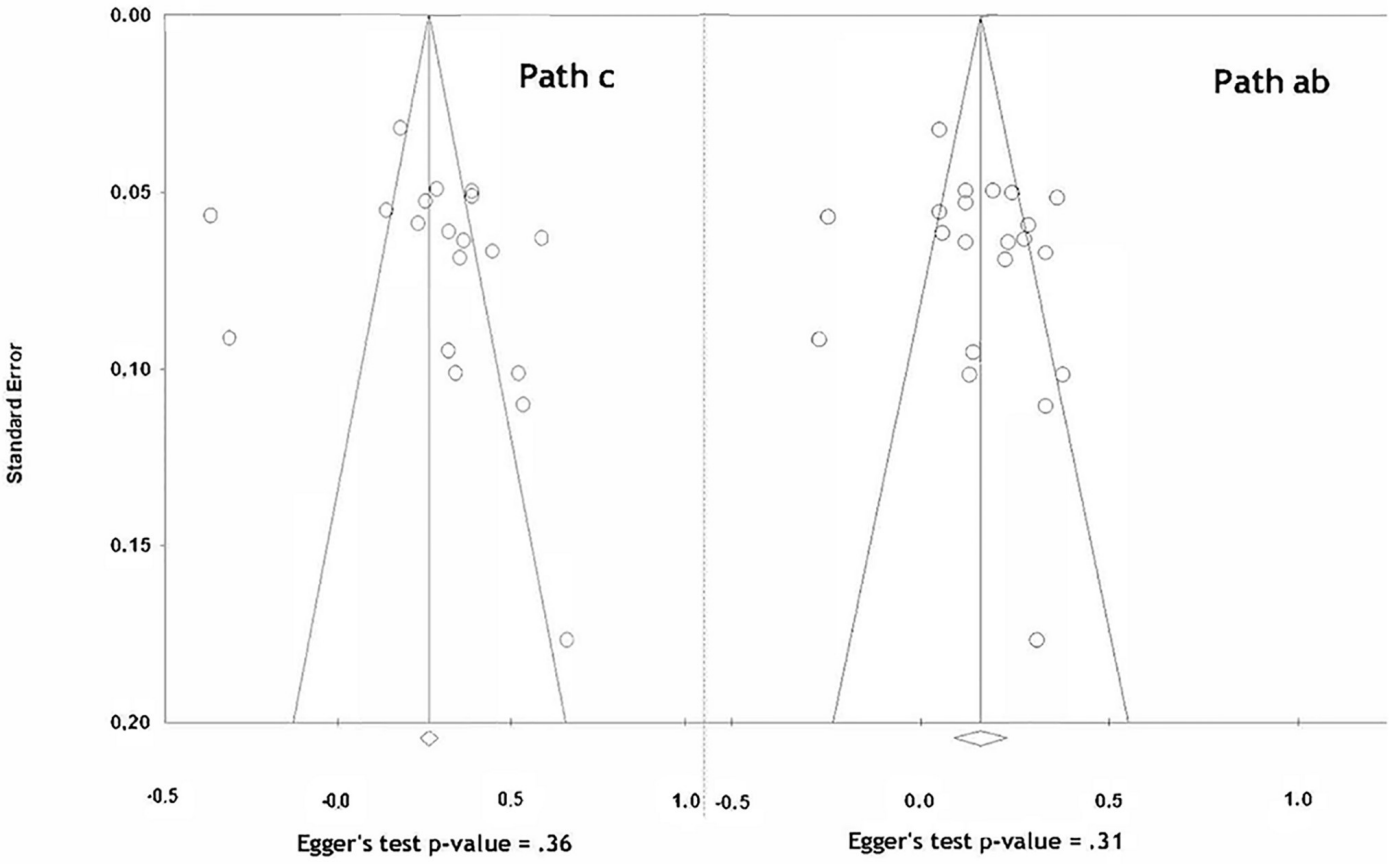
Funnel plots of correlations versus variance of correlations for total effect (path *c*) and indirect effect (path a*b). *Left hand side*: Funnel plot for the total effect (path *c*). *Right hand side*: Funnel plot for the indirect effect (path a*b). In both charts, Egger’s test p-value shows no publication bias (p > .05).

We assessed publication bias visually through funnel plots (Fig 2) and formal testing using Egger’s test [69]. We also performed sensitivity analyses, recalculating the pooled estimates under extreme conditions.

### Assessment of the quality of the studies

Eligible papers were evaluated for methodological quality with an adapted version for the present study of the critical appraisal tool developed by Lee et al. [38]. Further, four items were added in view of standard guidelines [70,71]. As a result, a checklist of nine items was obtained (S1 Appendix). The items included were as follows: clear description of objectives; appropriate design; representative sample; psychometric characteristics of the mediator and outcome variables reported; ascertain whether changes in the mediating variable preceded changes in the outcome variable; ascertain whether changes in the predictor variable preceded changes in the mediator variable; findings clearly described; and control for confounds. Each study was independently rated providing a score of 1 (yes) or 0 (no) to the 9 items. Studies were categorized into weak (scoring 0–4), moderate (scoring 5–7), and strong (scoring 8–9) based on these criteria. Disagreements between reviewers were resolved by discussion. Furthermore, we carried out a pooled analysis comparing low quality studies (scores < 5) with moderate-high quality studies (scores = > 5).

## Results

### Main characteristics of included studies in the review

Fig 1 summarizes the results of different stages of the systematic literature search. Initially, a total of 1655 records were selected as eligible to be screened by title and abstract, of which 110 were retrieved as potential relevant full-text and screened to determine eligibility. Among them, 86 did not meet the inclusion criteria and were excluded. Finally, 24 articles met inclusion criteria and were included for review, of them 21 were eligible for the meta-analysis.

As already noted, Tables 1 and 2 show the characteristics of each study. Regarding the design of the included studies, most of them adopted cross-sectional designs (n = 23) and only one used a longitudinal design [11]. Included studies were conducted in eleven different countries.

In reference to the characteristics of the sample, 14 studies used a community sample reported on adolescents (n = 2), university students (n = 8), and young adults (n = 4). Eight studies included both genders and six employed only women. Sample sizes ranged from 80 to 2644 participants and the mean age of participants across studies ranged from 12.9 to 39 years.

Eleven studies used clinical samples, including Pepping’s et al. [51] work referred to patients and three studies that combined samples of patients with EDs and healthy participants when performing the mediating analyses [59,61,63]. Three studies compared clinical and healthy samples. Five studies used exclusively samples with ED patients. Participants with ED were diagnosed using DSM-5 (n = 4), DSM-IV-TR (n = 3), or DSM-IV (n = 1). Two studies [44,59] did not report any criteria and one study relied on the self-informed diagnosis of the participant [62]. A total of 558 patients with a diagnosis of Anorexia Nervosa (AN), 568 of Bulimia Nervosa (BN), 143 of Binge Eating Disorder (BED) and 335 of Eating Disorder-Not Otherwise Specified (EDNOS) were included in the meta-analysis. Sample sizes ranged from 38 to 403 patients and their mean ages ranged from 21.17 to 39 years.

Attachment was assessed using the following self-report measures: The Attachment Style Questionnaire (ASQ) [72] (n = 4), a 40-item self-report questionnaire containing five scales: (a) adult secure attachment (via the Confidence scale), b) insecure anxious attachment (via the Need for approval and the Preoccupation with relationships scales), and (c) insecure avoidant attachment (via the Discomfort with closeness and Relationships as secondary scales); Adult Attachment Prototypes (AAP) [73] (n = 1), an instrument that assesses four categories of attachment described by Bartholomew and Horowitz [22]: secure, preoccupied, dismissing and fearful attachment; Adult Attachment scale (AAS) [74] (n = 2), an 18-item measure of attachment that yields scores for three adult attachment styles: secure (i.e., comfortable with closeness and independence in relationships), avoidant (i.e., prefer emotional distance from other people), and resistant (i.e., prefer closeness and worry about being abandoned); Cartes, Modèles Individuels de Relation (CaMir) [75] (n = 1), a questionnaire that measures insecure attachment assessing four dimensions (Preoccupation, Parental Interference, Self-Sufficiency, and Childhood Trauma); and Experiences in Close Relationships (ECR) [76] (n = 16) (including original version, revised versions or version adapted to children) which contains 36 items distributed in two subscales: (a) attachment related anxiety (i.e., the extent to which people are insecure vs. secure about the availability and responsiveness of close person) and (b) attachment related avoidance (i.e., the extent to which people are uncomfortable being close to others vs. secure depending on others). Furthermore, as attachment figure, studies assessed mother (n = 2); mother and father (n = 3); romantic partner (n = 3); parents, peers and romantic partner (n = 1) and the other studies referred to other close relationships (n = 15).

The main mediators explored in the reviewed literature were the following: emotional dysregulation (n = 5), body dissatisfaction (n = 4), social comparison (n = 3), perfectionism (n = 3), depressive symptoms (n = 2), mindfulness (n = 2) and neuroticism (n = 2). Additionally, other personality variables such as extraversion (n = 1) and narcissism (n = 1) and other mechanisms such as hope (n = 1), fear of abandonment (n = 1), family functioning (n = 1), appearance-based rejection sensitivity (n = 1), sensitivity to punishment (n = 1) and specific psychological ED traits (i.e., ineffectiveness, perfectionism, interpersonal distrust, interoceptive awareness, and maturity subscales taken from EDI-2) (n = 1) were tested as mediators; however, results regarding these variables were inconclusive due to the reduced sample size.

The main ED symptoms involved as the outcome of the mediation models included in this study were the following: bulimic symptoms (n = 7), diet (n = 5), body dissatisfaction (n = 3), binge eating (n = 1), interoceptive awareness EDI-2 (n = 1), impulsivity EDI-2 (n = 1), drive for thinness EDI-2 (n = 4), eating problems (n = 3), disordered eating (n = 2), ED symptoms (n = 5), eating pathology (n = 1), and eating behavioral problems (n = 1).

The methodological quality of the 24 retrieved studies ranked from 3 (weak) to 8 (strong) (see Table 3 for individual study quality ratings). The pooled estimate for the indirect effect of mediating variables was 62% and 53% for low quality studies and for moderate-high quality studies, respectively. In consequence, no significant differences in our results were found regarding quality.

**Table 3:** Quality assessment of the included studies.

	Aim clear	Design appropriate to aim	Sample representative	Psychometric characteristics	Acceptable methods of data analysis	Changes in M preceded changes in Y	Changes in X preceded changes in M	Clear findings	Control confounding factors	Final Rating
Dakanalis et al. [11]	1	1	0	1	1	1	1	1	1	**8**
Boone [28]	1	1	0	1	1	0	0	1	1	**6**
Bäck [42]	1	1	0	0	0	0	0	0	1	**3**
Bamford & Halliwell [43]	1	1	0	1	1	0	0	1	0	**5**
Tasca et al. [44]	1	1	0	1	1	0	0	1	1	**6**
Tasca et al. [45]	1	1	0	1	1	0	0	0	0	**4**
Eggert et al. [46]	1	1	0	1	0	0	0	1	0	**4**
Han et al. [47]	1	1	0	1	1	0	0	1	1	**6**
Kiang et al. [48]	1	1	0	1	1	0	0	1	0	**5**
Koskina et al. [49]	1	1	0	1	0	0	0	1	1	**5**
McDermott et al. [50]	1	1	1	1	1	0	0	1	0	**6**
Pepping et al. [51]	1	1	0	1	1	0	0	1	0	**5**
Schembri et al. [52]	1	1	1	1	0	0	0	1	0	**5**
Shanmugan et al. [53]	1	1	1	1	1	0	0	1	1	**7**
Ty et al. [54]	1	1	0	1	1	0	0	1	0	**5**
Van Durme et al. [55]	1	1	1	1	1	0	0	1	1	**7**
Dakanalis et al. [56]	1	1	0	1	1	0	0	1	1	**6**
De Paoli et al. [57]	1	1	0	1	1	0	0	1	1	**6**
De Paoli et al. [58]	1	1	0	1	1	0	0	1	1	**6**
Jakovina et al. [59]	1	1	0	1	0	0	0	1	1	**5**
Monteleone et al. [60]	1	1	1	1	1	0	0	1	0	**6**
Monteleone et al. [61]	1	1	0	1	1	0	0	1	0	**5**
Münch et al. [62]	1	0	0	1	1	0	0	1	0	**3**
Redondo & Luyten [63]	1	1	0	1	1	0	0	1	0	**5**

### Meta-analysis

The pooled correlation coefficients for path *a*, path *b*, total effect and indirect effect, with their CIs, the I^2^ statistic and effect sizes of each mediation model (*mediation ratio*) are presented in Table 4.

**Table 4:** Random effects pooled correlation coefficients of path a, path b, indirect effect and total effect; heterogeneity and mediation ratio.

	N° of models	Path *a* (95% CI)	*I*^*2*^	Path *b* (95% CI)	*I*^*2*^	Path *c* (95%CI)	*I*^*2*^	Path a*b (95%CI)	*I*^*2*^	|a*b/c|
**Total**	21	0.32 (0.13–0.49)	0.98	0.34 (0.19–0.47)	0.97	0.28 (0.20–0.37)	0.92	0.17 (0.10–0.23)	0.85	0.61
Clinical sample	6	-0.08 (-0.52–0.40)	0.99	0.04 (-0.41–0.34)	0.98	0.10 (-0.20–0.38)	0.96	0.01 (-0.18–0.20)	0.90	0.10
Non-clinical sample	14	0.45 (0.28–0.59)	0.97	0.45 (0.34–0.55)	0.95	0.34 (0.27–0.40)	0.80	0.20 (0.14–0.26)	0.75	0.59
Anxious	15	0.34 (0.12–0.53)	0.98	0.34 (0.15–0.50)	0.98	0.31 (0.21–0.40)	0.92	0.17 (0.10–0.24)	0.84	0.55
Avoidant	12	0.11 (-0.06–0.27)	0.96	0.20 (0.02–0.37)	0.97	0.22 (0.14–0.30)	0.85	0.09 (0.03–0.15)	0.70	0.41
High quality	9	0.20 (-0.12–0.48)	0.99	0.22 (0.01–0.41)	0.93	0.15 (-0.01–0.30)	0.95	0.08 (-0.04–0.20)	0.92	0.53
Low quality	12	0.41 (0.16–0.60)	0.98	0.42 (0.20–0.60)	0.97	0.37 (0.31–0.43)	0.65	0.23 (0.17–0.28)	0.51	0.62
Females only	15	0.34 (0.08–0.55)	0.98	0.38 (0.17–0.56)	0.98	0.33 (0.22–0.44)	0.93	0.19 (0.10–0.27)	0.83	0.58
**Dysfunctional ER**	5	0.33 (-0.24–0.73)	0.99	0.31 (-0.01–0.57)	0.98	0.21 (-0.06–0.45)	0.97	0.15 (-0.06–0.35)	0.95	0.71
Clinical	1	-0.64 (-0.70 − -0.57)	--	-0.37 (-0.46 − -0.27)	--	-0.35 (-0.44 − -0.25)	--	-0.24 (-0.34 − -0.13)	--	0.69
Non clinical	3	0.52 (0.001–0.81)	0.99	0.42 (0.24–0.57)	0.93	0.30 (0.16–0.43)	0.86	0.21 (0.01–0.40)	0.93	0.70
**Depressive symptoms**	2	0.49 (0.34–0.61)	0.80	0.52 (0.41–0.62)	0.70	0.35 (0.20–0.48)	0.73	0.25 (0.12–0.57)	0.64	0.71
Clinical	0	--	--	--	--	--	--	--	--	
Non clinical	2	0.49 (0.34–0.61)	0.80	0.52 (0.41–0.62)	0.70	0.35 (0.20–0.48)	0.73	0.25 (0.12–0.57)	0.64	0.71
**Body dissatisfaction**	4	0.35 (0.25–0.44)	0.42	0.53 (0.26–0.72)	0.94	0.31 (0.21–0.41)	0.46	0.18 (0.05–0.30)	0.62	0.58
Clinical	2	0.28 (0.19–0.36)	0	0.34 (0.10–0.53)	0.80	0.31 (0.22–0.40)	0	0.08 (-0.02–0.18)	0	0.26
Non clinical	2	0.43 (0.34–0.52)	0	0.67 (0.60–0.72)	0	0.39 (0.001–0.68)	0.81	0.28 (0.18–0.38)	0	0.72
**Neuroticism**	2	0.83 (0.10–0.98)	0.99	0.43 (0.30–0.55)	0.43	0.52 (0.44–0.59)	0	0.28 (0.18–0.38)	0	0.54
Clinical	0	--	--	--	--	--	--	--	--	
Non clinical	2	0.83 (0.10–0.98)	0.99	0.43 (0.30–0.55)	0.43	0.52 (0.44–0.59)	0	0.28 (0.18–0.38)	0	0.54
**Perfectionism**	3	0.35 (0.15–0.53)	0.92	0.38 (0.28–0.46)	0.71	0.27 (0.14–0.39)	0.81	0.14 (0.03–0.25)	0.72	0.52
Clinical	1	0.51 (0.43–0.58)	--	0.46 (0.38–0.53)	--	0.37 (0.28–0.45)	--	0.24 (0.15–0.33)	--	0.65
Non clinical	2	0.26 (0.08–0.43)	0.85	0.33 (0.26–0.39)	0	0.21 (0.07–0.35)	0.74	0.09 (0.02–0.16)	0	0.43
**Mindfulness**	2	-0.37 (-0.45–0.29)	0	-0.34 (-0.42–0.25)	0	0.27 (0.18–0.35)	0	0.12 (0.03–0.21)	0	0.44
Clinical	1	-0.33 (-0.55–0.07)	--	-0.46 (-0.64–0.22)	--	0.42 (0.31–0.52)	--	0.15 (-0.13–0.40)	--	0.36
Non clinical	1	-0.40 (-0.53–0.25)	--	-0.32 (-0.46–0.17)	--	0.37 (0.22–0.50)	--	0.13 (-0.03–0.29)	--	0.35
**Social comparison**	3	0.02 (-0.43–0.45)	0.97	0.27 (-0.47–0.79)	0.99	0.14 (-0.24–0.48)	0.95	0.03 (-0.22–0.28)	0.86	0.21
Clinical	1	-0.49 (-0.61 − -0.34)	--	-0.53 (-0.65 − -0.39)	--	-0.30 (-0.45 − -0.13)	--	-0.26 (-0.42 − -0.09)	--	0.87
Non clinical	2	0.28 (0.19–0.36)	0	0.61 (0.24–0.83)	0.98	0.35 (0.26–0.42)	0	0.17 (0.07–0.26)	0.16	0.49

*Note*. Path *a* = association between independent variable and mediator; Path *b* = association between mediator and dependent variable; Path *c* = total effect of the independent variable on the dependent variable; a*b = the indirect effect of the independent variable on the dependent variable controlling the mediator; *I*^*2*^ = heterogeneity; |a*b/c| = mediation ratio, effect size in mediation analysis.

#### Primary analyses

Overall, 61% of the total effect of insecure attachment on eating disorder symptoms was explained by the indirect effect of the main mediating variables (i.e., maladaptive emotion regulation strategies, depressive symptoms, body dissatisfaction, neuroticism, perfectionism, mindfulness and social comparison). Except for the subgroup of studies of low quality and those subgroups which included only two studies (such as mindfulness and neuroticism), heterogeneity was substantial overall and similarly high after stratification by sample, type of attachment or female sample. No individual study seemed to represent an influential point that increased heterogeneity dramatically. We, therefore, focused on the random effects analyses as recommended by experts and presented the fixed effects results for comparison purposes only (S3 Table).

Comparing sample types, in non-clinical samples the percentage of the total effect explained by the indirect effect was larger (59%) than in clinical samples (10%). Regarding attachment style, the percentage of the total effect explained by the indirect effect was 55% with anxious attachment and 41% with avoidant attachment. Additionally, the effect size of the mediating variables did not significantly differ by gender since the inclusion of men in the sample yielded similar results (61%) to the studies with female-only samples (58%).

#### Subgroup analysis by mediators

**Maladaptive emotion regulation**. Overall, the percentage of the total effect explained by the indirect effect was 71% [44,47,54,55,59]. The pooled estimates of the indirect effect of maladaptive emotion regulation strategies were significant (70%) in studies with non-clinical population [47,54,55]. The only study conducted with clinical sample found that maladaptive emotion regulation strategies did significantly mediate the relationship between insecure attachment and ED symptoms [44]. The percentage of the total effect explained by the indirect effect was 69%.

**Depressive symptoms**. The pooled estimate for the indirect effect of depressive symptoms was significant and large among non-clinical population [52,53]. The percentage of the total effect explained by the indirect effect was 71%. No study evaluated this model among patients with ED.

**Body dissatisfaction**. Overall, the percentage of the total effect explained by the indirect effect was 58%. The pooled estimates of the indirect effect of body dissatisfaction were significant in studies with non-clinical population (72%) [42,49]. However, body dissatisfaction did not significantly mediate the relationship between insecure attachment and ED symptoms in two studies with clinical samples [45,60]; 26% of the total effect that was explained by the indirect effect.

**Neuroticism**. The pooled estimate for the indirect effect of neuroticism was significant among non-clinical population [46,62]. The 54% of the total effect was explained by its indirect effect. No study evaluated this model among patients with ED.

**Perfectionism**. Overall, the 52% of the total effect was explained by the indirect effect of maladaptive perfectionism. The pooled estimate for the indirect effect of perfectionism was significant in studies with non-clinical sample [28,53], 43% of the total effect was explained by its indirect effect. The only study using a sample with ED patients found that perfectionism was a significant mediator between insecure attachment and ED symptoms among a sample with ED patients [56]. Specifically, the 65% of the total effect was explained by its indirect effect.

**Mindfulness**. Overall, 44% of the total effect was explained by the indirect effect [51,63]. Among non-clinical samples, 35% of the total effect was explained by its indirect effect [51]. Mindfulness was also a significant mediator between insecure attachment and ED symptoms among patients with ED [63]. The percentage of the total effect that was explained by the indirect effect was 36%.

**Social comparison**. Overall, the percentage of the total effect that was explained by the indirect effect was 21%. The pooled estimate for the indirect effect of social comparison was significant in studies with non-clinical sample [43,54]. The 49% of the total effect was explained by its indirect effect. The only study conducted with ED patients found that social comparison was a significant mediator between insecure attachment and ED symptoms [57]. The percentage of the total effect that was explained by the indirect effect was 87%.

**Additional mediators**. Some studies reported results on other mediators between insecure attachment style and ED symptoms. The number of studies was too low (less than 2) to be included in a meta-analysis. Therefore, these results are displayed in S4 Table. Only one longitudinal study identified *narcissism* as mediator (explaining 63% of eating disorder symptoms) among a large mixed sample [11]. E*xtraversion and family functioning* were found to mediate between insecure attachment and eating symptoms in women with ED, the total effect explained by the indirect effect was 43% and 66% respectively [62]. Furthermore, the proportion of the total effect that was explained by the indirect effect of other mediating variables such as *hope and specific psychological ED traits* were 21% and 37% in non-clinical mixed and female samples [48,50].

Finally, *appearance based rejection sensitivity* explained 89% of the symptoms of patients with ED; *sensitivity to punishment* and *fear of abandonment* explained 60% and 48%, respectively, of the ED symptoms in both clinical and non-clinical female groups [57,58,61].

#### Publication bias

For the total effect (path *c*), the Egger’s test yielded a *P* value of 0.36. Further, the Trim and Fill method suggested that seven studies might be missing. Without these seven potential studies, the pooled *r* was 0.28 (95%CI 0.20–0.37). Adding the seven suggested studies, the pooled *r* was 0.20 (95%CI 0.11–0.28).

Similarly, for the indirect effect (path a*b), there was no sign of publication bias since the Egger’s test yielded a *P* value of .31. The Trim and Fill method suggested adding four studies. Without these four potential studies, the pooled *r* was 0.17 (95%CI 0.10–0.23). With the four added studies, the pooled *r* was 0.11 (95%CI 0.04–0.18).

These analyses suggest that the observed effects are not meaningfully modified by potential publication bias.

#### Sensitivity analysis

To further evaluate the possibility that our results could be due to publication bias, we recalculated our pooled estimates under the following extreme assumptions: (1) published studies are only half of the studies identifying mediating variables between insecure attachment and ED symptoms, (2) all unpublished studies found an *r* of 0, (3) the unpublished studies have a sample size that is the same as the sample average of the published studies. Under these extreme assumptions, the pooled *r* for path *c* was still significant [0.14 (95%CI 0.08–0.20)]. Similarly, the pooled *r* for path a*b showed significance [0.08 (95%CI 0.04–0.12)]. As such, these analyses indicate that it is highly unlikely that the observed effects could have been undermined by publication bias.

## Discussion

The aim of this study was to provide a review and meta-analysis of studies investigating the mediators connecting insecure attachment with eating psychopathology at both clinical and sub-clinical level. Our results build on the already existent evidence that insecure attachment and ED symptoms could be explained by the indirect effect of various mechanisms. Consistent with previous reviews [5,8] and partly with our hypothesis, the mediators with the largest effect size were emotional dysregulation at both clinical and sub-clinical level, and depressive symptoms at sub-clinical level. In addition, body dissatisfaction, neuroticism, perfectionism, mindfulness and social comparison yielded significant impact also, but their effect size ranged from moderate to low.

An important contribution of the present study is the meta-analytic evidence of the influence that the aforementioned mediators have, regardless of gender or type of insecure attachment. In fact, current research tends to include male subjects, as insecurely attached men are also a group at risk of presenting disordered eating [77,78]. Likewise, results of previous reviews did not reveal conclusive data on the existence of an association between specific attachment patterns and ED subtypes [2,3]. For instance, while some authors [79,80] have found an association between avoidant attachment and AN, and anxious attachment and BN, others claim that most people with ED have either an avoidant attachment [81] or an anxious insecure attachment [82]. In light of these findings, further research should perform mediating analyses distinguishing between different diagnoses of ED, as the indirect effect of specific mediating mechanisms might be different regarding the nature of the symptomatology (e.g., emotion dysregulation might have a greater impact on bulimic symptoms).

Unexpectedly, we found that effect sizes were larger in general population than in ED patients. These results are in controversy with the broad literature highlighting the higher prevalence of insecure attachment in clinical samples than in non-clinical samples [3–6]. This result may be due, at least in part, to the fact that other mediating variables could better explain the relation between insecure attachment and ED symptoms, or even the direct effect of insecure attachment on ED symptoms may be stronger than the indirect effect through mediators in clinical samples of this meta-analysis. In addition, it should be noted the low number of studies with clinical samples. Further research is needed to ascertain this unclear finding.

This review underlines *dysfunctional emotion regulation* as the most robust nexus involved in the relationship between insecure attachment and ED symptoms at both clinical and sub-clinical level. Our findings agree with previous meta-analyses reporting moderate to large effect size of maladaptive emotion regulation strategies, either suppressing or accentuating emotional responses, on ED symptoms [7,21]. As such, this mechanism emerges as an intermediate pathway that links insecure attachment and ED symptomatology [44,47,54,55,59]. On the one hand, strategies used to express and to regulate emotions might be determined by the type of attachment [17,18,83] and, on the other hand, ED symptoms could be the manifestation of a difficulty in identifying emotions and an attempt to counteract such discomfort [84,85]. Consequently, insecurely attached people could either turn to binge eating as a way of getting distracted from adverse emotions [17,33], or to excessive exercise or dieting to reduce unwanted negative thoughts that very often follow binge eating [47,86].

As regards to the relative contribution of *depressive symptoms*, our findings indicate one of the highest effect sizes. Note that these results were based on studies with non-clinical samples, and therefore they are only tentative at best to acknowledge the effect on ED. A broad review [87] has concluded that maladaptive cognitive patterns and self-representations arising from negative early attachment experiences place individuals at-risk to develop depressive symptomatology following adverse life events. Related to emotion regulation, previous research suggested that anomalous eating behaviors emanate as a way to self-regulate or escape from negative emotions [33,34,88]. Moreover, depressive symptomatology has been identified as strong predictor of eating problems [12,89,90]. In the present meta-analysis, we found that individuals with insecure attachment think and behave in a manner that favors the development of eating psychopathology through depressive symptoms [52,74]. These results are in line with the dual pathway model of Stice [91] which provided prospective evidence of the mediational role of negative affect along with dieting in the subsequent development of bulimic pathology.

Our findings regarding *body dissatisfaction* suppose a further step that demonstrates the intermediate role of this variable in the relationship between insecure attachment and ED symptomatology [42,70]. From this perspective, individuals with insecure attachment could be particularly prone to internalize certain aesthetic standards, such as thinness in women or musculature in men, in the pursuit of acceptance and social approval [92,93]. When these goals are not achieved, body dissatisfaction increases, which in turn triggers a heightened risk for abnormal eating behaviors [92,94,95]. Against expectations, in our meta-analysis, the indirect effect of insecure attachment on ED symptoms through body dissatisfaction yielded not significant effect size among ED samples. It is possible that this result can be explained, at least partially, due to the characteristics of two out of four included studies. Thus, the model tested by Tasca [45], specifically designed for patients with BN, was applied to heterogeneous sample of women with ED mostly with AN. In addition, in this study [45] the authors simultaneously analyzed the negative affect which could have reduced the effect of body dissatisfaction. Moreover, another included work did not properly assess body dissatisfaction but the lived corporeality, i.e. the difficulty experiencing the body from an inner perspective [60]. In view of these considerations and the prominent risk that body dissatisfaction appears to carry to EDs [91,96], further research should examine its mediational impact in clinical samples.

In reference to *neuroticism*, we identified a significant impact on the development of ED symptoms. Specifically, two studies found that insecurely attached individuals with more neurotic personality characteristics were more likely to display disordered eating symptoms [46,62]. These findings are consistent with a wide body of literature supporting the link between neuroticism and several clinical and sub-clinical syndromes, among others, eating disorders [97,98] and, inversely, with the positive association between secure attachment and low neuroticism [99]. However, these results should be interpreted with caution, since neuroticism represents an unspecific personality dimension that englobes a variety of facets such as negative affect, affective instability and anxiety, involved in the etiology of diverse mental disorders [97].

In the present meta-analysis, *maladaptive perfectionism* was found to be a significant mediator at both clinical and sub-clinical level. According to the previous literature, insecure attachment strongly predicts the development of maladaptive perfectionism [100–102]. Specifically, insecurely attached individuals are likely to be overly self-critical, and therefore, to using strategies, such as maladaptive perfectionism, to counter feelings of worthlessness and hopelessness [92,102]. In addition, it is well-known the connection between perfectionism and ED symptoms in both clinical and non-clinical samples [56,103,104]. Consequently, people with insecure attachment are prone to seek excellent standards difficult to reach, frequently concerning body shape and weight and its control [19,92,105], which favors the appearance of unhealthy eating behaviors to achieve this unrealistic aims [28,53,56].

Reduced *mindfulness* capacity (i.e., inability to be fully aware of current experience or present reality) [106] was also identified as one of the mediating mechanisms [51,63]. This result is not surprising as the associations between insecure attachment and mindfulness [106–108] and between mindfulness and eating pathology [109,110] have been previously established. Mindfulness capacity in insecurely attached individuals might be indeed compromised as they tend to engage in cognitive and emotional processes, such as worrying about a future abandonment (anxious attachment) or refusing to attend to an emotion or need (avoidant attachment), that are contrary to mindful states [108]. This is especially relevant for patients with bulimia and binge eating who often struggle detecting and discriminating interoceptive cues (i.e., hunger and satiety versus one’s inner feelings) [60].

Finally, *social comparison* was identified as a significant mediator in non-clinical and clinical samples, but presented the lowest effect size; therefore, more research is required to confirm this finding. Insecurely attached people develop dysfunctional beliefs about oneself [19], that conducted themselves to excessively compare to others whom they deemed potentially better to assess their self-image, which in turn promote the risk for developing abnormal eating attitudes [43,54,57].

## Limitations of the present study

The present study has several limitations worth noting. First, due to the scarce number of studies in relation to some mediators, only a subset of the studies could be included in this meta-analysis. Second, the majority of the findings proved in this meta-analysis were provided by normal population (predominantly younger Caucasian women), thus reducing the possibility for generalization; caution is needed in extrapolating the results to other sociodemographic groups. Third, the inclusion of cross-sectional data in the present meta-analysis did not make it possible to draw definitive conclusions regarding the development of the ED psychopathology and reflects the need for future studies to implement prospective designs. Fourth, some of the included studies [42,49,52,59] used exclusively Sobel test to prove the significance of mediation despite the fact that this test has been overcome [65]. Fifth, the heterogeneity of effects between studies was high. Nevertheless, as it is claimed by experts, heterogeneity in a meta-analysis should be viewed as expected rather than inevitable and not as a nuisance [111]. Therefore, we decided to interpret our results taking random-effects estimates as it might be the most appropriate way to deal with this issue [112]. Sixth, the *mediation ratio* [67] was used as a summary of the effect size for each mediator despite the fact that it is a measure that suffers from several limitations [35,37]. However, as the data included in the present meta-analysis reported larger total effects than the indirect effects and of the same sign, we followed recommendations of its application [35]. In addition, it should be noted that so far it is the most widely used measure of effect size for mediation models and a method relatively unaffected by sample size. Finally, most studies relied only on self-reports. Hence, some response biases may have affected the results.

Overall, the results of this meta-analysis should be interpreted with caution, and only in the context of the characteristics from included studies. Nevertheless, as far as we know, this study is the first meta-analysis exploring the effect sizes of mediators that connect insecure attachment and eating symptoms in both clinical and sub-clinical samples at any age.

## Implications for research

Future studies should explore prospectively mediating mechanisms implementing designs with a temporal sequence ascertaining the precedence of the independent variable on the mediator and, by the same token, of the mediator on the dependent variable [38]. Because of the possible relationship between the main mediators found in this review, it would be interesting to explore the interplay among them through sequential multiple mediation models and moderated mediation models to understand the contribution of each mediator. Further, future mediation studies should apply powerful statistical techniques such as SEM with bootstrapping in order to strengthen conclusions and reporting the magnitude of the mediated effect [65]. Additionally, given the paucity of studies, it will be useful in the future to ascertain more precisely whether the mediational effects of such variables differ by specific diagnoses of ED and also by age distribution. Lastly, the control for confounding variables merits particular attention to rule out possible spurious effects [70].

## Clinical implications

A consistent finding in the literature is the influence of insecure attachment on the poorer therapeutic outcomes among patients with ED [13,113]; as insecurely attached patients tend to change or to abandon therapy frequently [45,114]. As suggested by Tasca and Balfour [5], clinicians should assess patient’s quality and level of attachment in order to adapt the therapy and to guarantee better therapeutic outcomes.

Our meta-analytic results suggest that insecure attachment patterns along with others psychological variables may be important targets of clinical interventions of ED; hence, it is recommended that clinicians include therapeutic strategies focused, not only on the quality of attachment, but also on the mediators that maintain and aggravate eating symptomatology or pose a risk for their possible development [115]. Adopting this strategy could improve ED treatment outcomes and reduce drop-out rates [116]. For instance, intervening at the level of self-representations by improving confidence and to provide skills to better manage negative emotions and interpersonal problems may decrease the distress and subsequent symptoms of eating disorder such as binge eating, purging or extreme exercise or dieting [13]. Additionally, the combination of a cognitive-behavioral treatment with mindfulness techniques could be a new path of treatment that could offer promising results [51,110], as it has been already tested in patients with major depressive disorder [117].

Moreover, given the impact of insecure attachment on the later development of eating pathology, early interventions that decrease the onset of EDs are of critical importance. For instance, providing parents with the skills necessary to enhance their children’s capacity for self-regulating when facing challenging situations [87]. Also, school-based prevention programs for children and adolescents including modules of psychoeducation addressing, not only eating pathology and its risks, but also management of negative emotions, coping with interpersonal problems, confronting rigid and unrealistic standards and the promotion of a healthy self-image, might be important to consider [118].

## Conclusions

The results obtained in the present meta-analysis extend previous findings by showing that emotion dysregulation, depressive symptoms, body dissatisfaction, neuroticism, perfectionism, mindfulness and social comparison could be essential psychological mechanisms for explaining the pathways through which insecure attachment may increase the vulnerability to eating symptoms. However, since the application of mediation analysis in disordered eating research is still in its early stages, more studies are needed to corroborate our results. Specifically, longitudinal studies are required to clarify the interplay of the mediators between insecure attachment and ED symptoms.

## Supporting information

S1 Appendix Checklist for measuring study quality. (DOCX)

S1 Table PRISMA checklist. (DOC)

S2 Table Detailed extracted and coded data for meta-analysis. (DOCX)

S3 Table Fixed effects pooled correlation coefficients of path a, path b, indirect effect and total effect; heterogeneity and mediation ratio. (DOCX)

S4 Table Meta-analysis results for additional mediators. (DOCX)

S1 Fig Forest plot path c and random effects summary. (TIFF)

S2 Fig Forest plot path a*b and random effects summary. (TIFF)
