# Supplementary material for: Mediators linking insecure attachment to eating symptoms: A systematic review and meta-analysis
Source: PLoS One. 2019 Mar 7;14(3):e0213099. doi: 10.1371/journal.pone.0213099 (PMC6405186; doi:10.1371/journal.pone.0213099)
Supplement: S1 Appendix — (DOCX) [file pone.0213099.s001.docx]

**S1 Appendix. Checklist for measuring study quality**

| **Items** | **Yes** | **No** |
| --- | --- | --- |
| 1. Is there hypothesis/aim/objective of the study clearly described? (Objectives are formulated adequately: precise, clear and comprehensive) |  |  |
| 1. Is the study design appropriate to objectives? |  |  |
| 1. Is the study sample representative? (Participants are recruited from a representative setting that relates to the studies aims and hypotheses) |  |  |
| 1. Were the psychometric characteristics of the mediator and outcome variables reported? (Computed from the present study or a reference provided) |  |  |
| 1. Were statistically appropriate/ acceptable methods of data analysis used? (This includes the product of coefficient approach with bootstrapped confidence intervals, structural equation modelling, latent growth modelling, and causal mediation analysis) |  |  |
| 1. Did the study ascertain whether changes in the mediating variable preceded changes in the outcome variable? |  |  |
| 1. Did the study ascertain whether changes in the predictor variable preceded changes in the mediator variable? |  |  |
| 1. Are the main findings of the study clearly described? (Simple outcome data should be reported for all major findings so that the reader can check the major analysis and conclusions) |  |  |
| 1. Did the study control for possible confounding factors? (Variables that may impact on results are identified and controlled for in terms of statistical analysis) |  |  |
