# Supplementary material for: Mediators linking insecure attachment to eating symptoms: A systematic review and meta-analysis
Source: PLoS One. 2019 Mar 7;14(3):e0213099. doi: 10.1371/journal.pone.0213099 (PMC6405186; doi:10.1371/journal.pone.0213099)
Supplement: S2 Table — (DOCX) [file pone.0213099.s003.docx]

**S2 Table. Detailed extracted and coded data for meta-analysis**

| **Author/s (Year)/Country** | **Sample** | **Gender** | **Clinical vs**  **Non-clinical** | **IV** | **Mediator** | **DV** | **Path *a*** | **Path *b*** | **Path *c*** | | ***a*b*** | **Quality rating** |
| --- | --- | --- | --- | --- | --- | --- | --- | --- | --- | --- | --- | --- |
| Bäck (2011)/Sweden | 35 | 1 | 0 | Fearful | BD | Eating problems | 0.42 | 0.72 | 0.58 | 0.30 | | 3 |
| Koskina & Giovazolias (2010)/Greece | 381 | 1 | 0 | Anxious | BD | Dietary symptoms | 0.45 | 0.70 | 0.24 | 0.32 | | 5 |
| Koskina & Giovazolias (2010)/Greece | 381 | 1 | 0 | Anxious | BD | Bulimia symptoms | 0.45 | 0.62 | 0.22 | 0.28 | | 5 |
| Koskina & Giovazolias (2010)/Greece | 100 | 0 | 0 | Anxious | BD | Dietary symptoms | 0.24 | 0.62 | 0.24 | 0.15 | | 5 |
| Monteleone et al. (2017)/Italy | 113 | 1 | 1 | Avoidant | BD | EDI-2-Interoceptive awareness | 0.30 | 0.49 | 0.33 | 0.15 | | 6 |
| Monteleone et al. (2017)/Italy | 113 | 1 | 1 | Avoidant | BD | EDI-2-Impulsivity | 0.30 | 0.40 | 0.29 | 0.12 | | 6 |
| Tasca et al. (2006)/Canada | 268 | 1 | 1 | Anxious | BD | Drive for thinness | 0.31 | 0.39 | 0.40 | 0.12 | | 4 |
| Tasca et al. (2006)/Canada | 268 | 1 | 1 | Anxious | BD | Dietary restraint | 0.31 | 0.05 | 0.29 | 0.02 | | 4 |
| Tasca et al. (2006)/Canada | 268 | 1 | 1 | Avoidant | BD | Drive for thinness | 0.23 | 0.39 | 0.31 | 0.09 | | 4 |
| Tasca et al. (2006)/Canada | 268 | 1 | 1 | Avoidant | BD | Dietary restraint | 0.23 | 0.05 | 0.25 | 0.01 | | 4 |
| Bamford & Halliwell (2009)/UK | 213 | 1 | 0 | Anxious | SC | EDI-Drive for thinness | 0.30 | 0.74 | 0.36 | 0.22 | | 5 |
| Bamford & Halliwell (2009)/UK | 213 | 1 | 0 | Anxious | SC | EDI-Body dissatisfaction | 0.30 | 0.74 | 0.30 | 0.22 | | 5 |
| Bamford & Halliwell (2009)/UK | 213 | 1 | 0 | Anxious | SC | EDI-Bulimia | 0.30 | 0.74 | 0.35 | 0.22 | | 5 |
| Ty & Francis (2013)/Australia | 247 | 1 | 0 | Anxious | SC | Disordered eating | 0.27 | 0.44 | 0.37 | 0.12 | | 5 |
| Ty & Francis (2013)/Australia | 247 | 1 | 0 | Avoidant | SC | Disordered eating | 0.24 | 0.44 | 0.32 | 0.11 | | 5 |
| De Paoli et al. (2017b)/Australia | 122 | 2 | 1 | Avoidant | SC | Disordered eating | -0.49 | -0.53 | 0.30 | 0.26 | | 6 |
| Boone (2013)/Belgium | 328 | 2 | 0 | Avoidant(mother) | P | EDI-2-Bulimia | 0.13 | 0.31 | 0.13 | 0.04 | | 6 |
| Boone (2013)/Belgium | 328 | 2 | 0 | Avoidant(father) | P | EDI-2-Bulimia | 0.20 | 0.28 | 0.15 | 0.05 | | 6 |
| Dakanalis et al. (2013)/Italy | 403 | 1 | 1 | Anxious | P | ED symptoms | 0.48 | 0.46 | 0.31 | 0.22 | | 6 |
| Dakanalis et al. (2013)/Italy | 403 | 1 | 1 | Avoidant | P | ED symptoms | 0.54 | 0.46 | 0.43 | 0.25 | | 6 |
| Shanmugan, Jowett & Meyer (2012)/UK | 411 | 2 | 0 | Anxious | P | Eating pathology | 0.43 | 0.35 | 0.38 | 0.15 | | 7 |
| Shanmugan, Jowett & Meyer (2012)/UK | 411 | 2 | 0 | Avoidant | P | Eating pathology | 0.26 | 0.35 | 0.18 | 0.09 | | 7 |
| Han & Pistole (2014)/US | 381 | 2 | 0 | Insecure | ER | Binge eating | 0.79 | 0.46 | 0.37 | 0.35 | | 6 |
| Tasca et al. (2009)/Canada | 310 | 1 | 1 | Anxious | ER | ED symptoms | -0.64 | -0.37 | 0.35 | 0.24 | | 6 |
| Ty & Francis (2013)/Australia | 247 | 1 | 0 | Anxious | ER | Disordered eating | 0.48 | 0.53 | 0.37 | 0.25 | | 5 |
| Ty & Francis (2013)/Australia | 247 | 1 | 0 | Avoidant | ER | Disordered eating | 0.39 | 0.53 | 0.32 | 0.21 | | 5 |
| Van Durme, Braet & Goossens (2015)/Belgium | 952 | 2 | 0 | Anxious | ER | Restraint eating | 0.29 | 0.22 | 0.17 | 0.06 | | 7 |
| Van Durme, Braet & Goossens (2015)/ Belgium | 952 | 2 | 0 | Anxious | ER | Eating problems concerns | 0.29 | 0.30 | 0.26 | 0.09 | | 7 |
| Van Durme, Braet & Goossens (2015)/ Belgium | 952 | 2 | 0 | Avoidant | ER | Restraint eating | 0.07 | 0.22 | 0.15 | 0.02 | | 7 |
| Van Durme, Braet & Goossens (2015)/Belgium | 952 | 2 | 0 | Avoidant | ER | Eating problems concerns | 0.07 | 0.30 | 0.13 | 0.02 | | 7 |
| *Jakovina et al. (2018)/ Croatia | 100 | 1 | 2 | Anxious | ER | Bulimic symptoms | 0.64 | 0.57 | 0.48 | 0.36 | | 5 |
| Eggert, Levendosky & Klump (2007)/US | 85 | 1 | 0 | Anxious | N | ED symptoms | 0.94 | 0.34 | 0.49 | 0.32 | | 4 |
| Münch, Hunger & Schweitzer (2016)/ Germany | 253 | 1 | 0 | Insecure | N | ED symptoms | 0.56 | 0.48 | 0.53 | 0.27 | | 3 |
| Shanmugan, Jowett & Meyer (2012)/UK | 411 | 2 | 0 | Anxious | D | Eating pathology | 0.58 | 0.47 | 0.38 | 0.27 | | 7 |
| Shanmugan, Jowett & Meyer (2012)/UK | 411 | 2 | 0 | Avoidant | D | Eating pathology | 0.23 | 0.47 | 0.18 | 0.11 | | 7 |
| Schembri & Evans (2008)/Australia | 225 | 1 | 0 | Anxious | D | Bulimic symptoms | 0.56 | 0.58 | 0.42 | 0.32 | | 5 |
| Pepping et al. (2015)/ Australia | 144 | 1 | 0 | Anxious | M | Eating pathology | -0.39 | -0.32 | 0.41 | 0.12 | | 5 |
| Pepping et al. (2015)/ Australia | 144 | 1 | 0 | Avoidant | M | Eating pathology | -0.41 | -0.32 | 0.32 | 0.13 | | 5 |
| Pepping et al. (2015)/ Australia | 55 | 1 | 1 | Anxious | M | Eating pathology | -0.38 | -0.46 | 0.26 | 0.17 | | 5 |
| Pepping et al. (2015)/ Australia | 55 | 1 | 1 | Avoidant | M | Eating pathology | -0.27 | -0.46 | 0.22 | 0.12 | | 5 |
| *Redondo & Luyten (2018)/ Spain | 361 | 1 | 2 | Anxious | M | Dieting | -0.38 | -0.38 | 0.31 | 0.14 | | 5 |
| Redondo & Luyten (2018)/ Spain | 361 | 1 | 2 | Anxious | M | Bulimia | -0.38 | -0.37 | 0.3 | 0.14 | | 5 |
| Redondo & Luyten (2018)/ Spain | 361 | 1 | 2 | Anxious | M | Oral control | -0.38 | -0.23 | 0.25 | 0.09 | | 5 |
| Redondo & Luyten (2018)/ Spain | 361 | 1 | 2 | Avoidant | M | Dieting | -0.38 | -0.38 | 0.3 | 0.14 | | 5 |
| Redondo & Luyten (2018)/ Spain | 361 | 1 | 2 | Avoidant | M | Bulimia | -0.38 | -0.37 | 0.28 | 0.14 | | 5 |
| Redondo & Luyten (2018)/ Spain | 361 | 1 | 2 | Avoidant | M | Oral control | -0.38 | -0.23 | 0.23 | 0.09 | | 5 |
| Redondo & Luyten (2018)/ Spain | 361 | 1 | 2 | Avoidant | M | Dieting | -0.35 | -0.38 | 0.24 | 0.13 | | 5 |
| Redondo & Luyten (2018)/ Spain | 361 | 1 | 2 | Avoidant | M | Bulimia | -0.35 | -0.37 | 0.23 | 0.13 | | 5 |
| Redondo & Luyten (2018)/ Spain | 361 | 1 | 2 | Avoidant | M | Oral control | -0.35 | -0.23 | 0.15 | 0.08 | | 5 |

*Note.* Gender: 0 = Males, 1 = Females, 2 = Both; Clinical = Coded as 1; Clinical and Non-Clinical = 2; IV = Insecure attachment style; Mediator: BD = Body Dissatisfaction, SC = Social Comparison, P = Perfectionism; ER = Maladaptive emotion regulation; N = Neuroticism; D = Depressive symptoms; M = Mindfulness; DV = Dependent variable; Path *a* = association between independent variable and mediator; Path *b* = association between mediator and dependent variable; Path *c =* total effect of the independent variable on the dependent variable; *a*b* = the indirect effect of the independent variable on the dependent variable controlling the mediator.

*These studies were only computed for the total effect and could not be stratified by type of sample as they combined clinical and non-clinical samples to test the mediational effects.
