# Supplementary material for: Mediators linking insecure attachment to eating symptoms: A systematic review and meta-analysis
Source: PLoS One. 2019 Mar 7;14(3):e0213099. doi: 10.1371/journal.pone.0213099 (PMC6405186; doi:10.1371/journal.pone.0213099)
Supplement: S3 Table — (DOCX) [file pone.0213099.s004.docx]

**S3 Table. Fixed effects pooled correlation coefficients of path a, path b, indirect effect and total effect; heterogeneity and mediation ratio**

|  | **Nº of models** | **Path a**  **(95% CI)** | **I^2^** | **Path b**  **(95% CI)** | **I^2^** | **Path c**  **(95%CI)** | **I^2^** | **Path a*b**  **(95%CI)** | **I^2^** | **\|a*b/c\|** |
| --- | --- | --- | --- | --- | --- | --- | --- | --- | --- | --- |
| **Total** | 21 | 0.29 (0.27-0.31) | 0.98 | 0.32 (0.30-0.35) | 0.97 | 0.26 (0.24-0.28) | 0.92 | 0.15 (0.12-0.17) | 0.85 | 0.58 |
| Clinical sample | 6 | 0.01 (-0.04-0.07) | 0.99 | 0.08 (0.02-0.13) | 0.98 | 0.11 (0.06-0.17) | 0.96 | 0.02 (-0.03-0.08) | 0.90 | 0.18 |
| Non clinical sample | 14 | 0.39 (0.37-0.42) | 0.97 | 0.42 (0.40-0.45) | 0.95 | 0.30 (0.27-0.33) | 0.80 | 0.18 (0.15-0.21) | 0.75 | 0.60 |
| Anxious | 15 | 0.29 (0.26-0.32) | 0.98 | 0.32 (0.29-0.35) | 0.98 | 0.28 (0.25-0.31) | 0.92 | 0.15 (0.12-0.18) | 0.84 | 0.54 |
| Avoidant | 12 | 0.15 (0.12-0.18) | 0.96 | 0.26 (0.23-0.29) | 0.97 | 0.23 (0.19-0.25) | 0.85 | 0.09 (0.06-0.12) | 0.70 | 0.39 |
| High quality | 9 | 0.27 (0.24-0.30) | 0.99 | 0.28 (0.24-0.31) | 0.93 | 0.19 (0.15-0.22) | 0.95 | 0.10 (0.07-0.12) | 0.92 | 0.53 |
| Low quality | 12 | 0.32 (0.29-0.36) | 0.98 | 0.39 (0.36-0.43) | 0.97 | 0.36 (0.32-0.39) | 0.65 | 0.21 (0.18-0.25) | 0.51 | 0.58 |
| Females only | 15 | 0.26 (0.23-0.29) | 0.98 | 0.35 (0.32-0.38) | 0.98 | 0.29 (0.26-0.32) | 0.93 | 0.17 (0.14-0.21) | 0.83 | 0.59 |
| **Dysfunctional ER** | 5 | 0.26 (0.22-0.30) | 0.99 | 0.26 (0.22-0.30) | 0.98 | 0.17 (0.13-0.22) | 0.97 | 0.10 (0.06-0.15) | 0.95 | 0.59 |
| Clinical | 1 | -0.64 (-0.70 - -0.57) | -- | -0.37 (-0.46 - -0.27) | -- | -0.35 (-0.44 - -0.25) | -- | -0.24 (-0.34 - -0.13) | -- | 0.69 |
| Non clinical | 3 | 0.42 (0.37-0.46) | 0.99 | 0.36 (0.31-0.40) | 0.93 | 0.25 (0.21-0.30) | 0.86 | 0.15 (0.11-0.20) | 0.93 | 0.60 |
| **Depressive symptoms** | 2 | 0.47 (0.41-0.53) | 0.80 | 0.51 (0.45-0.57) | 0.70 | 0.33 (0.26-0.40) | 0.73 | 0.24 (0.16-0.31) | 0.64 | 0.73 |
| Clinical | 0 | -- | -- | -- | -- | -- | -- | -- | -- |  |
| Non clinical | 2 | 0.47 (0.41-0.53) | 0.80 | 0.51 (0.45-0.57) | 0.70 | 0.33 (0.26-0.40) | 0.73 | 0.24 (0.16-0.31) | 0.64 | 0.73 |
| **Body dissatisfaction** | 4 | 0.35 (0.28-0.41) | 0.42 | 0.49 0.43-0.54) | 0.94 | 0.29 (0.22-0.36) | 0.46 | 0.18 (0.10-0.25) | 0.62 | 0.62 |
| Clinical | 2 | 0.28 (0.19-0.36) | 0 | 0.30 (0.20-0.39) | 0.80 | 0.31 (0.22-0.40) | 0 | 0.08 (-0.02-0.18) | 0 | 0.26 |
| Non clinical | 2 | 0.43 (0.34-0.52) | 0 | 0.67 (0.60-0.72) | 0 | 0.27 (0.17-0.37) | 0.81 | 0.28 (0.18-0.38) | 0 | 1.04 |
| **Neuroticism** | 2 | 0.72 (0.66-0.77) | 0.99 | 0.45 (0.36-0.53) | 0.43 | 0.52 (0.44-0.59) | 0 | 0.28 (0.18-0.38) | 0 | 0.54 |
| Clinical | 0 | -- | -- | -- | -- | -- | -- | -- | -- |  |
| Non clinical | 2 | 0.72 (0.66-0.77) | 0.99 | 0.45 (0.36-0.53) | 0.43 | 0.52 (0.44-0.59) | 0 | 0.28 (0.18-0.38) | 0 | 0.54 |
| **Perfectionism** | 3 | 0.36 (0.31-0.41) | 0.92 | 0.38 (0.33-0.43) | 0.71 | 0.27 (0.22-0.33) | 0.81 | 0.14 (0.09-0.20) | 0.72 | 0.52 |
| Clinical | 1 | 0.51 (0.43-0.58) | -- | 0.46 (0.38-0.53) | -- | 0.37 (0.28-0.45) | -- | 0.24 (0.15-0.33) | -- | 0.65 |
| Non clinical | 2 | 0.27 (0.20-0.34) | 0.85 | 0.33 (0.26-0.39) | 0 | 0.22 (0.15-0.29) | 0.74 | 0.09 (0.02-0.16) | 0 | 0.41 |
| **Mindfulness** | 2 | -0.37 (-0.45--0.29) | 0 | -0.34 (-0.42--0.25) | 0 | 0.27 (0.18-0.35) | 0 | 0.12 (0.03-0.21) | 0 | 0.44 |
| Clinical | 1 | -0.33 (-0.55--0.07) | -- | -0.46 (-0.64--0.22) | -- | 0.42 (0.31-0.52) | -- | 0.15 (-0.13-0.40) | -- | 0.36 |
| Non clinical | 1 | -0.40 (-0.53--0.25) | -- | -0.32 (-0.46--0.17) | -- | 0.37 (0.22-0.50) | -- | 0.13 (-0.03-0.29) | -- | 0.35 |
| **Social comparison** | 3 | 0.12 (0.03-0.20) | 0.97 | 0.40 (0.33-0.47) | 0.99 | 0.22 (0.14-0.29) | 0.95 | 0.08 (-0.004-0.16) | 0.86 | 0.36 |
| Clinical | 1 | -0.49 (-0.61 - -0.34) | -- | -0.53 (-0.65 - -0.39) | -- | -0.30 (-0.45 - -0.13) | -- | -0.26 (-0.42 - -0.09) | -- | 0.87 |
| Non clinical | 2 | 0.28 (0.19-0.36) | 0 | 0.60 (0.54-0.66) | 0.98 | 0.35 (0.26-0.42) | 0 | 0.17 (0.08-0.26) | 0.16 | 0.49 |

*Note*. Path *a* = association between independent variable and mediator; Path *b*= association between mediator and dependent variable; Path *c =* total effect of the independent variable on the dependent variable; *a*b* = the indirect effect of the independent variable on the dependent variable controlling the mediator; *I^2^* = heterogeneity; |*a*b/c*| = mediation ratio, effect size in mediation analysis.
