# Supplementary material for: Mediators linking insecure attachment to eating symptoms: A systematic review and meta-analysis
Source: PLoS One. 2019 Mar 7;14(3):e0213099. doi: 10.1371/journal.pone.0213099 (PMC6405186; doi:10.1371/journal.pone.0213099)
Supplement: S4 Table — (DOCX) [file pone.0213099.s005.docx]

**S4 Table. Meta-analysis results for additional mediators**

| **Author/s (Year)/Country** | **Sample** | **Gender** | **Clinical vs**  **Non-clinical** | **IV** | **Mediator** | **DV** | **Path *a*** | **Path *b*** | **Path *c*** | | ***a*b*** | **Quality rating** |
| --- | --- | --- | --- | --- | --- | --- | --- | --- | --- | --- | --- | --- |
| Münch, Hunger & Schweitzer (2016)/Germany | 253 | 1 | 0 | Insecure | E | ED symptoms | -0,49 | -0,47 | 0,53 | 0,23 | | 3 |
| Münch, Hunger & Schweitzer (2016)/Germany | 253 | 1 | 0 | Insecure | FF | ED symptoms | -0,61 | -0,57 | 0,53 | 0,35 | | 3 |
| McDermott et al. (2015)/US | 2644 | 1 | 0 | Anxious | H | Eating problems | -0,27 | -0,15 | 0,27 | 0,04 | | 6 |
| McDermott et al. (2015)/US | 2644 | 1 | 0 | Avoidant | H | Eating problems | -0,18 | -0,15 | 0,08 | 0,03 | | 6 |
| Kiang & Harter (2006)/US | 146 | 1 | 0 | Anxious (mother) | EDPT | Eating behavioral problems | 0,22 | 0,27 | 0,14 | 0,06 | | 5 |
| Kiang & Harter (2006)/US | 146 | 1 | 0 | Anxious (partner) | EDPT | Eating behavioral problems | 0,23 | 0,27 | 0,31 | 0,06 | | 5 |
| Kiang & Harter (2006)/US | 146 | 1 | 0 | Avoidant(mother) | EDPT | Eating behavioral problems | 0,35 | 0,27 | 0,12 | 0,1 | | 5 |
| De Paoli et al. (2017b)/Australia | 122 | 2 | 1 | Anxious | ARS | EDI-Drive for thinness | 0,52 | 0,61 | 0,32 | 0,32 | | 6 |
| De Paoli et al. (2017b)/Australia | 122 | 2 | 1 | Anxious | ARS | EDI-Body dissatisfaction | 0,52 | 0,69 | 0,43 | 0,36 | | 6 |
| De Paoli et al. (2017a)/Australia | 508 | 1 | 0 | Anxious | FA | ED symptoms | 0,5 | 0,28 | 0,34 | 0,14 | | 6 |
| De Paoli et al. (2017a)/Australia | 108 | 1 | 1 | Anxious | FA | ED symptoms | 0,6 | 0,46 | 0,4 | 0,28 | | 6 |
| Dakanalis et al. (2016)/Italy | 2055 | 2 | 0 | Anxious | Na | Bulimic symptoms | 0,27 | 0,26 | 0,12 | 0,07 | | 8 |
| Dakanalis et al. (2016)/Italy | 2055 | 2 | 0 | Avoidant | Na | Dietary symptoms | 0,29 | 0,29 | 0,12 | 0,08 | | 8 |
| Monteleone et al. (2018)/ Italy | 123 | 1 | 2 | Anxious | SP | EDI-Drive for thinness | 0,39 | 0,42 | 0,28 | 0,16 | | 5 |
| Monteleone et al. (2018)/ Italy | 123 | 1 | 2 | Anxious | SP | EDI-Body dissatisfaction | 0,41 | 0,34 | 0,22 | 0,14 | | 5 |

*Note.* Gender: 0 = Males, 1 = Females, 2 = Both; Clinical = Coded as 1; Clinical and Non-Clinical = 2; IV = Insecure attachment style; Mediator:E = Extraversion; FF = Family Functioning; H = Hope; EDPT = Eating Disorder Psychological Traits; ARS = Appearance-based Rejection Sensitivity; FA = Fear of Abandonment; Na = Narcissism; SP = Sensitivity to Punishment; DV = Dependent variable; Path *a* = association between independent variable and mediator; Path *b* = association between mediator and dependent variable; Path *c =* total effect of the independent variable on the dependent variable; *a*b* = the indirect effect of the independent variable on the dependent variable controlling the mediator.
