# Supplementary figures and images for: Mediators linking insecure attachment to eating symptoms: A systematic review and meta-analysis
Source: PLoS One. 2019 Mar 7;14(3):e0213099. doi: 10.1371/journal.pone.0213099 (PMC6405186; doi:10.1371/journal.pone.0213099)

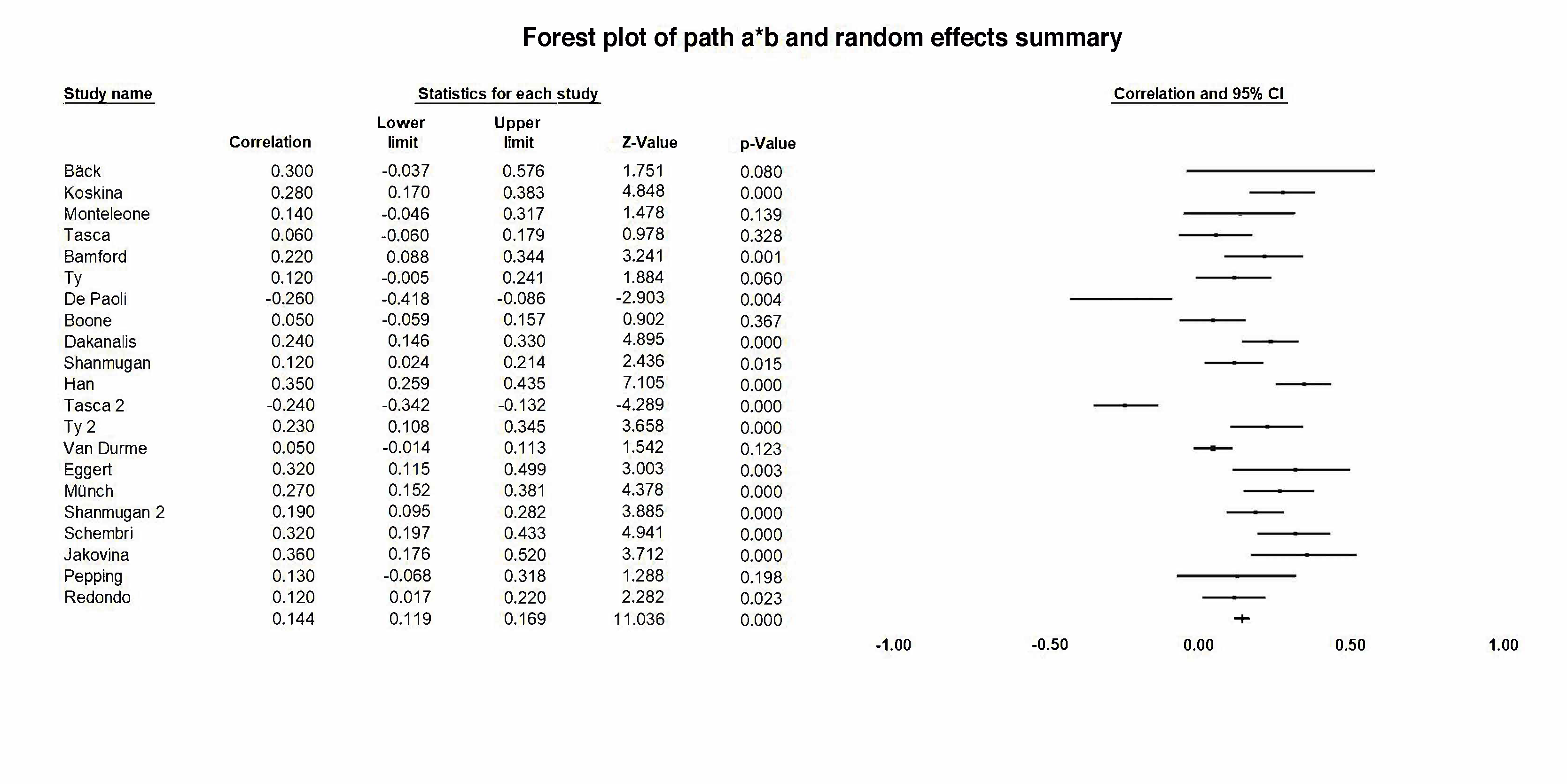

Supplement: S2 Fig — (TIFF) [file pone.0213099.s007.tiff]
